# Comparative Influence of Dendron and Dicarboxylate Coatings on the Hyperthermia Performances of Cubic and Spherical Magnetic Nanoparticles

**DOI:** 10.3390/ijms26199324

**Published:** 2025-09-24

**Authors:** Cristian Iacovita, Constantin Mihai Lucaciu, Barbara Freis, Céline Kiefer, Sylvie Bégin-Colin

**Affiliations:** 1 Department of Pharmaceutical Physics-Biophysics, Faculty of Pharmacy, Iuliu Hatieganu University of Medicine and Pharmacy, 6 Pasteur St., 400349 Cluj-Napoca, Romania; cristian.iacovita@umfcluj.ro; 2 UMR CNRS-UdS 7504, Institut de Physique et Chimie des Matériaux, CNRS, Université de Strasbourg, 23 Rue du Loess, BP 43, 67034 Strasbourg, France; bcdfreis@gmail.com (B.F.); celine.kiefer@ipcms.unistra.fr (C.K.); sylvie.begin@ipcms.unistra.fr (S.B.-C.); 3 UMR CNRS-UdS 7515, Institut de Chimie et Procédés pour l’Energie, l’Environnement et la Santé, CNRS, Université de Strasbourg, 25 Rue du Becquerel, BP 43, 67087 Strasbourg, France

**Keywords:** magnetic nanoparticles, dendrons, hyperthermia, dipolar interactions

## Abstract

Surface functionalization of magnetic nanoparticles, commonly used for their biocompatibility in biomedical applications, plays a critical role in optimizing iron oxide nanoparticles (IONPs) for magnetic hyperthermia (MH), a promising modality in cancer therapy. In this study, we provide the first comprehensive comparison of hyperbranched dendron coatings versus linear dicarboxylate ligands on IONPs, revealing their contrasting impacts on heating efficiency under varying magnetic field amplitudes (H). Dendron-coated IONPs outperform dicarboxylate-coated ones at low fields (H < 25 kA/m) due to reduced dipolar interactions and enhanced Brownian relaxation. Conversely, dicarboxylate coatings excel at high fields (H > 25 kA/m) by enabling magnetically aligned chains, which amplify hysteresis losses. Our work also introduces an approach to dynamically modulate the heating efficiency of IONPs by applying a static DC magnetic field (H_DC_) in conjunction with the alternating magnetic field (AMF). We observed a coating-dependent response to H_DC_ in the parallel configuration (H_DC_ aligned with AMF), the specific absorption rate (SAR) increased by ~620 W/g_Fe_ for cubes and ~370 W/g_Fe_ for spheres at high AMF amplitudes (H > 30 kA/m) for dicarboxylate-coated IONPs. This enhancement arises from magnetically aligned chains (visualized via Transmission Electron Microscopy), which amplify extrinsic anisotropy and hysteresis losses; in contrast, for dendron-coated IONPs, their SAR values decreased under H_DC_ (up to ~665 W/g_Fe_ reduction for cubes in the perpendicular configuration), as the thick dendron shell prevents close interparticle contact, suppressing chain formation and fanning rotation modes. These findings underscore the significance of surface functionalization in enhancing the therapeutic efficacy of magnetic nanoparticles.

## 1. Introduction

Cancer treatment can gain advantages from exploiting the therapeutic impacts achieved by precisely managing temperature within tissues. Indeed, elevating the temperature within tumor tissues (to 43 °C and above), a phenomenon known as hyperthermia, is employed as an additional approach for fighting cancer in clinical scenarios, often in combination with chemotherapy or radiotherapy [1,2]. The advances in the synthesis of iron oxide magnetic nanoparticles (MNPs) [3,4,5,6] have promoted the development of hyperthermia techniques, offering highly localized heating by activating MNPs using external magnetic fields (referred to as magnetic hyperthermia—MH) [7,8,9,10] or near-infrared (NIR) light (referred to as photothermia) [11,12,13,14] leading to successful reduction of tumors. Besides local therapy, MNPs can also provide high-resolution imaging of tumors by magnetic resonance imaging (MRI) [15,16] and magnetic particle imaging (MPI) [17,18].

To render iron oxide MNPs suitable for medical applications, they need to undergo functionalization with a biocompatible coating. This coating must possess the capacity to fulfill a spectrum of essential functionalities such as preserving the integrity of the magnetic core, providing stable dispersion in solutions, promoting favorable biodistribution, preventing protein attachment, and enabling selective accumulation in tumor tissues while avoiding buildup in organs responsible for clearance, and enhancing cellular uptake. The spinel iron oxide core can be functionalized with various types of coatings, including both inorganic materials, such as silica [19] and gold [20], and organic compounds like small ligands/molecules, synthetic or natural polymers [21], and lipids [22]. A highly promising category of organic coatings has emerged over the last decade, known as dendrons, which possess intricate branching structures offering a multitude of functionalities [23]. The dendrons (Figure S1a) bear two phosphonate anchoring groups to ensure a strong attachment to the surface of MNPs, thanks to the robust iron-complexing ability exhibited by the -PO(OH)_2_ group [24]. In parallel, the integration of three peripheral PEG chains enhances biocompatibility, while the longer PEG chain, bearing a carboxylate group, holds the exciting prospect of enabling future coupling with various other therapeutic molecules or chromophores.

A series of in vitro and in vivo studies have demonstrated the antifouling properties of dendron coating, showcasing minimal accumulation within the reticuloendothelial system (RES) of dendron-coated MNPs [15,23,24,25,26,27]. Their favorable biodistribution and bioelimination profiles suggest strong potential for in vivo affinity targeting, making use of their intrinsic hyperthermia properties for targeted therapy [14,28]. Our investigations have evidenced that when systemically administered, dendron-coated MNPs coupled with a melanin-targeting ligand exhibit favorable biodistribution and biokinetics [29]. Their trajectory homes in on melanoma lesions precisely, wherein melanoma cells selectively internalize them [29]. Furthermore, dendron-coated MNPs functionalized with a metronidazole-based ligand, capable of discerning hypoxic tissues, stand out as promising candidates for advancing agents that identify such hypoxic tissues [30]. This advancement and recent ones [14,28] hold great promise in the realms of tumor treatment and in vitro tissue engineering. Thus, these dendronized IONPs hold the potential to orchestrate the specific accumulation of IONPs within tumoral cells through the strategic coupling of targeting ligands on their surface.

However, the MNPs displaying suitable heating values by MH are mostly spherical nanoparticles around 20 nm [30] or cubic-shaped MNPs [31] around 19 nm, or flower-shaped MNPs [32,33]. These MNPs thus display high saturation magnetization (M_s_) and magnetic anisotropy (K), leading to strong magnetostatic interactions between them. In the case of ferromagnetic nanoparticles, these interactions favor uncontrolled aggregation into large clusters, which significantly decreases their hyperthermia efficiency [34,35]. To overcome this, coatings with large chitosan molecules [36] or thick silica shells [37] have been employed, since they reduce dipolar interaction, improve colloidal stability, and thus enhance heating performances by approximately 1000 W/g_Fe_ compared with thinly coated MNPs. Similarly, long polyethylene molecules (20 kDa) attached to large MNPs facilitate a high heating capacity that can be maintained in various media such as distilled water, physiological saline solution, agar, and cell culture [38,39]. In contrast, for superparamagnetic (SP) nanoparticles the situation can be different: their ability to dynamically align under an AMF may increase heating efficiency, and in this case, excessive separation though very thick silica shells can actually reduce their SAR [40].

Considering the promising biomedical applications of dendron-coated IONPs, this work aims to investigate how dendron functionalization influences the heating performances of MNPs under MH conditions. To this end, we synthesized spherical and cubic magnetite nanoparticles via a modified thermal decomposition method (TD) and subsequently compared their heating efficiencies when coated with dendrons or with short linear dicarboxylate ligands. Using our dedicated magnetic hyperthermia setup, we evaluated the heating capacities of these MNPs across a wide range of AMF strengths, and we further explored how the application of a static bias magnetic field (H_DC_) modifies their heat dissipation. This approach allows us to better understand the role of surface functionalization and dipolar interactions in determining the MH performance of iron oxide NPs.

## 2. Results and Discussions

### 2.1. Structural Characterization of MNPs

Existing challenges in the synthesis of MNPs involve the achievement of well-regulated MNPs concerning their composition, dimensions, morphology, and crystalline structure. Initially pioneered by Hyeon and Sun, the TD synthesis technique emerged as the most fitting approach, captivating the interest of researchers due to its manifold benefits: (i) it enables the production of MNPs with a notably high yield; (ii) the MNPs are concurrently coated with a surfactant in situ, preventing them from aggregation, and displaying remarkable uniformity in size distribution; (iii) and it facilitates meticulous control of both size and shape attributes [41,42]. Iron acetylacetonate and iron oleate are the most frequently used iron precursors [42,43,44,45,46]. Nevertheless, in our research, we opted for self-produced iron stearates [47]. This choice was motivated by some aging issues with home-made iron oleate and significant impurities present in commercially available iron stearate precursors, which have a detrimental effect on the overall structural integrity of the resulting MNPs.

**Figure 1:**
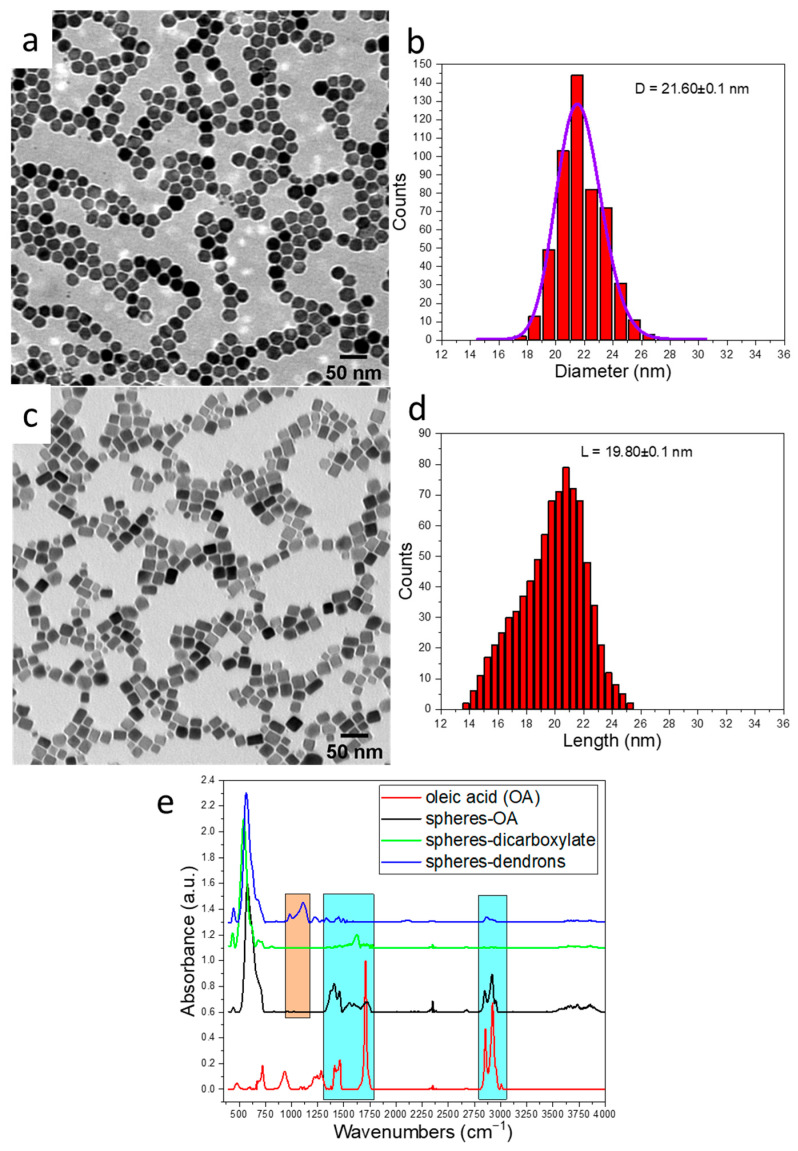
(**a**–**d**) TEM images of spheres and cubes and their corresponding size distribution histograms. The purple line is a log-normal fit of the size distribution. (**e**) FT-IR spectra of oleic acid, oleic acid-coated spherical MNPs, decarboxylated MNPs (upon oleic acid oxidation), and dendron-coated MNPs. The characteristic bands of oleic acid and dendrons are highlighted in blue and orange, respectively. The spectra are normalized to the highest absorption band and shifted upward for clarity. The FT-IR spectra of cubic MNPs looked similar and were omitted from the plot for the sake of simplicity.

Potential candidates for MH include spherical magnetite MNPs with an average size ranging from 20 to 25 nm [30], which falls within the boundary between the blocked monodomain and SP regimes. A significant limitation of the TD synthesis method is the formation of core–shell structures that have been frequently observed when producing MNPs larger than 15 nm of various shapes [48]. These structures consist of a wüstite core and a spinel shell (Fe_1−x_O@Fe_3−x_O_4_) [48]. This phenomenon often occurs because the nuclei generated during the TD process possess a wüstite composition, and the oxidation rate is comparatively sluggish compared to the growth rate. To enhance the oxidation kinetics of the wüstite phase during the growth phase, different synthesis protocols have been devised. We have thus optimized the synthesis protocol as detailed in reference [44], which consists briefly of introducing a small quantity of dibenzyl ether (DBE) into the reaction mixture and employing a low heating rate. Well-dispersed spherical MNPs with a spinel composition and an average diameter of 21.6 ± 0.1 nm (standard deviation of 1.5 nm), as depicted in Figure 1a,b, were thus obtained.

The shape anisotropy of the MNPs offers a significant advantage in enhancing their MH efficiency. Among the various shapes that have been studied, nanocubes exhibit particularly promising heating properties [25,49,50]. The formation of the nanocubes can be achieved through the use of specific shape-driving ligands like sodium oleate (NaOl) [51] by using dibenzyl ether [52], or by employing a high heating rate [53,54]. Here, nanocubes with a spinel composition and an average edge length of 19.8 ± 0.1 nm (standard deviation of 2.5 nm), as depicted in Figure 1c,d, have been synthesized by using a homemade iron stearate (FeSt_2_) precursor, a ratio NaOI/oleic acid (OA)—of 80:20, and a high heating rate.

The nanospheres and nanocubes have been coated by dendron molecules by a direct ligand exchange process, or the OA at their surface has been oxidized as described in the experimental part. These coating steps have been checked by the FTIR spectroscopy. FTIR spectrum of pure OA (displayed as the red spectrum in Figure 1e) exhibits a prominent peak at 1708 cm^−1^ corresponding to the asymmetric stretching of the -C = O bond [55]. There are two significant peaks at 2854 cm^−1^ and 2923 cm^−1^ representing the symmetric and asymmetric stretching of -CH_2_ groups of the alkyl chain, and additionally, two notable peaks at 1462 cm^−1^ and 1412 cm^−1^ associated with in-plane and out-of-plane stretching of O-H bonds [55].

Upon analyzing MNPs coated with OA (illustrated as the black spectrum in Figure 1e), a discernible shift towards lower frequencies is observed for all these peaks [56]. Particularly, the intense peak at 1708 cm^−1^ undergoes a significant reduction in intensity [56]. Notably, two fresh peaks emerge at 1595 cm^−1^ and 1558 cm^−1^, which are attributed to the asymmetric and symmetric stretching of -COO^−^ groups, respectively, and are indicative of the complexation between iron atoms and the carboxylate groups of OA [56]. This confirms that OA molecules are chemisorbed onto the surface of MNPs. Following the water transfer using sodium periodate-induced oxidation of OA the FT-IR spectrum (displayed as green spectrum in Figure 1e) reveals faintly discernible bands associated with OA. Notably, the peaks linked to the asymmetric and symmetric stretching vibrations of the carboxylic group experienced a shift to 1622 cm^−1^ and 1565 cm^−1^, respectively, providing evidence for the breakdown of the alkyl chain of OA during the oxidation process [56,57]. The intensity of the bands related to OA also experienced a significant decrease when the MNPs were coated with a dendron layer. Except for the Fe-O band at 566 cm^−1^, the FT-IR spectrum primarily showcases bands associated with the dendron coating. These include the C-O-C band around 1100 cm^−1^ and phosphonate bands ranging from 1100 to 800 cm^−1^, as indicated by the orange highlights in the blue spectrum in Figure 1e [14,24]. It is important to note that the predominant Fe-O bands in all samples closely resemble the expected IR spectrum of magnetite with a small shoulder indicating a slight surface oxidation. This confirms that the composition of all the samples is mainly consistent with magnetite [14]. Furthermore, when comparing the MNPs before and after being transferred into water, there were no observable alterations in their size and shape.

### 2.2. Magnetic Characterization of Dicarboxylate-Coated MNPs

**Figure 2:**
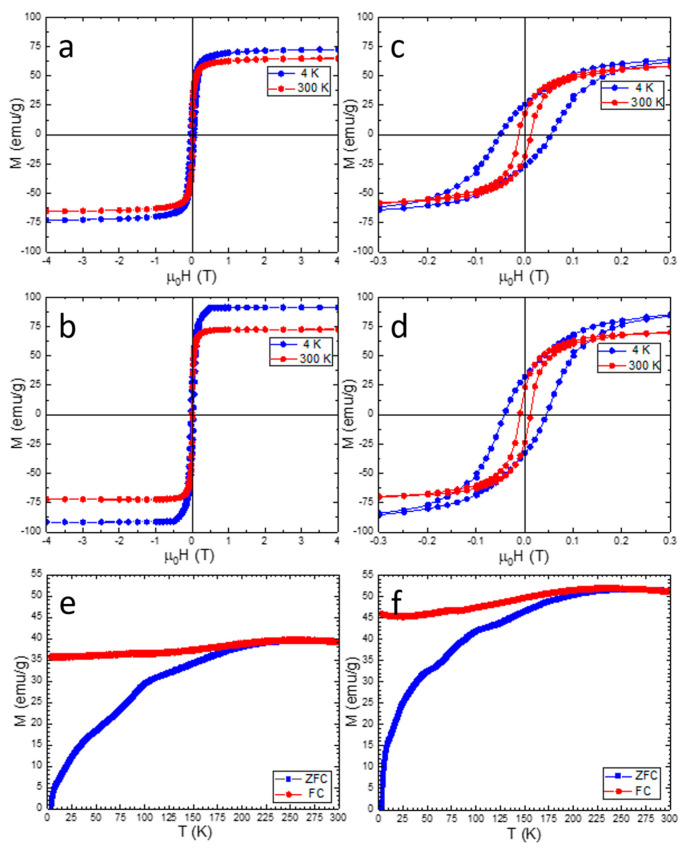
Magnetic hysteresis loops (wide field range and close-up field range) measured at 4 K and 300 K and zero-field cooling and field cooling magnetization curves for (**a**,**c**,**e**) spherical and (**b**,**d**,**f**) cubic dicarboxylated MNPs.

The magnetic properties of MNPs were studied by examining the magnetization as a function of the applied magnetic field (represented as M–H curves) at two temperatures, 4 K and 300 K. Additionally, temperature-dependent magnetization data were collected using two distinct measurement approaches: zero-field cooling (ZFC) and field cooling (FC) (Figure 2a–f). In the ZFC method, the samples were initially cooled to 4 K without any external magnetic influence, and subsequently, their magnetization was observed as the temperature increased while an external magnetic field of 4 kA/m (50 Oe) was applied. In contrast, the FC method involved cooling the samples in the specified external magnetic field. The key magnetic parameters, namely M_s_, coercivities (H_c_), and remanences (M_r_), for both samples are presented in Table 1. The magnetic properties were measured on dicarboxylated MNPs since the magnetic composition is preserved after dendronization thanks to the two anchoring phosphonate groups, which protect magnetite from further oxidation [25].

The MNPs demonstrate ferrimagnetic properties at the temperature of 4 K, as evidenced by the presence of open hysteresis loops in the M–H curves (see Figure 2c,d). Notably, the spherical MNPs exhibit a higher H_c_ compared to the cubic ones (Table 1). The cubic MNPs display an M_s_ of 91 emu/g, a value quite close to that of bulk magnetite at room temperature (92 emu/g). This similarity suggests a high level of crystallinity and magnetite composition homogeneity in the cubic MNP samples. In contrast, the spherical MNPs exhibit a lower M_s_ value of only 73 emu/g. At 300 K, both spherical and cubic MNPs display a reduction in saturation magnetization compared to low temperature values, consistent with thermal effects and surface spin disorder. The slight widening observed in the hysteresis loops (coercivity of 9 kA/m for both types of MNPs) indicates that the MNPs are not purely SP but are in an intermediate state between SP and ferrimagnetism, retaining a permanent magnetic moment at 300 K, as reflected by the presence of remanent magnetization (Table 1). Furthermore, at 300 K, both types of MNPs exhibit M_r_/M_s_ values below 0.5 (Table 1), indicating that they possess a uniaxial anisotropy.

The ZFC magnetization curves of both spherical and cubic MNPs display a broad maximum at approximately 265 K and 270 K, respectively (Figure 2e,f). Such broad features are typically associated with a wide distribution of energy barriers, which in this case arise not only from the finite size distribution (16–30 nm for spheres and 14–26 nm for cubes) but predominantly from strong dipolar interactions and collective dynamics within the nanoparticle assemblies. The nearly flat FC curves at low temperatures indicate collective behavior as well. The feature visible around 100 K in the ZFC curves of both types of MNPs can be assigned to the Verwey transition of magnetite, in agreement with FT-IR data confirming the magnetite phase of MNPs. Finally, the small shoulder around 40 K may be related to oxygen adsorption/freezing on the particle surface or to surface spin-glass-like effects.

### 2.3. Magnetic Hyperthermia Performances of MNPs

**Figure 3:**
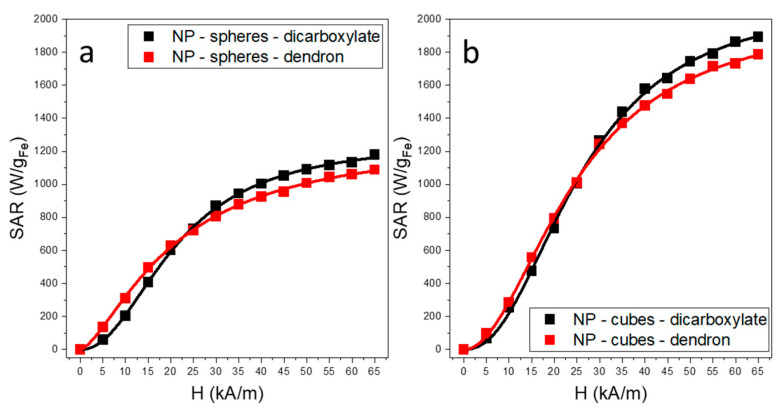
SAR dependence on the H for (**a**) spherical and (**b**) cubic MNPs with dicarboxylate and dendron coatings, dispersed in water at a concentration of 1 mg_Fe_/mL. The lines are the best fits using the logistic function.

The application of dendron coating is recognized for its ability to maintain a remarkably high colloidal stability of MNPs, while preserving their magnetic properties [23]. Nevertheless, the impact of this dendron coating on the MH performance of MNPs has not yet been reported. The comprehensive understanding of the MH characteristics of MNPs requires their analysis across a wide spectrum of magnetic field amplitude (H). The MH properties of the MNPs were thus evaluated using an AMF ranging from 5 to 65 kA/m, at a fixed frequency of 355 kHz. Figure 3 illustrates the evolution of SAR values with H, also referred to as specific loss power (SLP), for both spherical and cubic MNPs, with dicarboxylate and dendron coating.

At this point, we emphasize that we used a large range of AMF amplitudes to better understand the physical mechanisms of heat generation and to clearly evidence the sigmoidal dependence of SAR on H. From a medical perspective, both frequency and amplitude are limited by patient tolerance due to eddy current side effects. The Atkinson–Brezovich limit (H × f = 4.85 × 10^8^ A·m^−1^·s^−1^) was originally defined from discomfort reported in patients during MH therapy [58,59], while later studies suggested higher thresholds, up to 10–100 times larger [60]. More recent in vivo work on rats showed that values up to 9.59 × 10^9^ A·m^−1^·s^−1^ (≈27 kA/m in our setup) can still be safe [61]. Additionally, Tsipala et al. [62] demonstrated that using intermittent fields can mitigate eddy currents, allowing amplitudes up to ~38 kA/m (H × f = 1.35 × 10^10^ A·m^−1^·s^−1^). Other proposals include revised upper limits [63] or alternative metrics such as the “maximal SAR” of eddy current-induced tissue absorption [64]. For these reasons, we investigated MNP heating in a broad AMF range (5–65 kA/m), while keeping in mind biomedical applicability constraints.

In the MH literature, it is considered that in the case of SP-MNPs, the heat release mechanism involved is related to “relaxation losses” involving both the Brown relaxation due to the physical rotation of the MNPs and the Néel relaxation mechanism due to the magnetization rotation within an MNP. For ferromagnetic MNPs, the heat release is explained in terms of “hysteresis losses” as these particles present an open hysteresis curve characterized by a M_r_, and a coercive field, H_c_. However, as pointed out by Carrey et al. [65] and other authors, this separation between “relaxation losses” and “hysteresis losses” is somehow artificial, as the heat release in any MNPs could be explained by the dynamic hysteresis, and that, finally, all losses are hysteresis losses. Nevertheless, depending on external parameters, some authors identify a “magnetic regime” in which the heat release is dominated by the magnetization reversal (when the physical rotation of the MNPs is blocked) and a “viscous regime” during which the rotation of the MNP dominates the heat release.

At room temperature, our MNPs are ferrimagnetic and in a blocked state; therefore, their heating in AMF can be described by Stoner–Wohlfarth (SW) type models. In the ideal SW model, magnetization reversal occurs when the field amplitude equals the anisotropy field (H_k_ = 2 K/μ_0_M_s_, μ_0_ is the vacuum magnetic permeability, M_s_ is the saturation magnetization), producing a square hysteresis loop whose area is proportional to the anisotropy constant (K) and determines the SAR. Anisotropy thus controls both the maximum heat release and the minimum field amplitude required for significant heating (H_max_ ≥ 0.5 H_k_).

For MNPs with uniaxial anisotropy, magnetization reversal takes place when the external field overcomes the anisotropy barrier. While no exact analytical SAR expression exists for the ferromagnetic state, numerical simulations (e.g., Landau–Lifshitz–Gilbert) and empirical models show that thermal effects, particle concentration, and interparticle interactions reduce the hysteresis loop area compared to the ideal case [65]. Consequently, collective behavior, particle alignment, and dipolar interactions play a crucial role in determining the actual heating efficiency in MH.

As Figure 3 shows for both types of MNPs, the SAR dependence on H is sigmoidal, characterized by a steep increase at low H followed by saturation at H higher than a so-called hyperthermia coercive field H_cHyp_, representing the amplitude of the AMF corresponding to the highest slope in the SAR = f(H) curve.

We used a logistic function to fit the SAR dependence on H (refer to the Supplementary Information Section S3), which provides coefficients of determination R^2^ > 0.999. Three major parameters are derived from the fittings: SAR_max_, H_cHyp_, and the exponent n, which is related to the squareness of the dynamic hysteresis loop, showing how steep SAR’s evolution is with H (Table 2). For the dicarboxylated spheres, we obtained a SAR_max_ of 1285 W/g_Fe_ and an H_cHyp_ of 13.2 kA/m.

Spheric MNPs with dicarboxylate coating show SAR rising from 60 W/g_Fe_ at 5 kA/m to 602 W/g_Fe_ at 20 kA/m, then gradually saturate as H increases, corresponding to the transition from minor to major loops [66,67]. Dendronized MNPs exhibit higher SAR than dicarboxylate-coated MNPs at low H, rising from 135 W/g_Fe_ at 5 kA/m to 500 W/g_Fe_ at 15 kA/m and approaching the latter at 20–25 kA/m. Beyond 30 kA/m, SAR saturates slightly below dicarboxylate-coated values, with H_cHyp_ = 6.94 kA/m and SAR_max_ = 1270 W/g_Fe_ (Table 2). Cubic MNPs with short dicarboxylate layers show higher SAR than spherical ones (Figure 3b). The SAR difference is <100 W/g_Fe_ at low fields (5–15 kA/m) but rises to ~700 W/g_Fe_ at 65 kA/m. SAR exhibits a sigmoidal dependence on H, with H_cHyp_ = 17.05 kA/m and SAR_max_ = 2130 W/g_Fe_ (Table 2). When comparing SAR values, dendron-coated cubic MNPs show a trend similar to spherical ones (Figure 3a,b). Their SAR is slightly higher at low H but decreases as H increases (Figure 3b). Notably, for both spheres and cubes, dendron-coated MNPs outperform carboxylate-coated ones within medically safe H limits.

The differences in heat release by the two classes of MNPs are influenced not solely by the intrinsic properties of their magnetic core, but also by their coating, which should impact the magnetic interactions among individual MNPs [68,69]. Specifically, dipolar interactions between MNPs subjected to AMF can lead to the formation of chains or clusters with various two-dimensional or three-dimensional arrangements, significantly affecting their MH performances [70,71].

Previous experimental and theoretical studies highlighted the crucial role of MNP chains and columns formed under AMF when analyzing magnetic hyperthermia [72]. Magnetic interactions within these chains induce uniaxial anisotropy, enhancing hysteresis loop squareness and heating efficiency, although this competes with intrinsic magnetocrystalline anisotropy, particularly affecting low-anisotropy MNPs [72]. Under the AMF, MNPs tend to self-organize into well-defined chains [67,73,74,75,76,77]. This alignment introduces an additional extrinsic anisotropy along the chain direction [78], whose magnitude depends on dipolar interactions between neighboring particles and the number of MNPs within a chain [78,79]. Chain formation effectively increases the energy barrier along the field direction, enhancing the SAR for small to moderate chain sizes. However, as the chain grows or the structure evolves toward more cylindrical or 2D/3D assemblies, this effect diminishes, leading to a decrease in SAR [80].

The presence of dendron coating leads to an increased separation between MNPs, thereby reducing extrinsic anisotropy. In contrast, short dicarboxylate-coated MNPs come closer to each other in the presence of an AFM, facilitating the emergence of extrinsic anisotropy. This phenomenon could potentially explain the observation that the SAR of short dicarboxylate-coated MNPs equals and eventually surpasses that of dendron-coated MNPs as the H is gradually elevated to 65 kA/m.

From another point of view, the physical rotation of dendron-coated MNPs becomes more effortless compared to short-coated MNPs, leading to aggregation into clusters. As with dendronized MNPs of such size, the MH heating results more from the Brownian relaxation than the Neel relaxation [48], one may expect an enhanced contribution of Brownian relaxation for dendron-coated MNPs, making them heat up more effectively at low H in contrast to carboxylate-coated MNPs [40]. This outcome holds significant implications for biological applications, as the efficiency of MH for MNPs within cells is primarily influenced by MNP aggregation, before considering their confinement within intracellular vesicles [81].

One can notice that the difference in SAR values between dendron and dicarboxylate-coated MNPs is less significant in the case of cubic MNPs. This phenomenon can be rationalized by considering that dipolar interactions might drive the spatial arrangement of bare cubic MNPs in small chains when subjected to an AMF. As a result, these compact assemblies could increase the Neel relaxation, compensating for the reduction in Brownian relaxation.

Based on the above results, it seems that the presence of the dendron coating reduces the strength of dipolar interactions among MNPs [82]. This results in a distinct magnetic heating behavior at low and high H ranges compared to the dicarboxylate-coated MNPs. To further explore the dendron coating’s impact on SAR, an additional series of MH experiments was conducted, which involved applying a static DC magnetic field (H_DC_) alongside the AMF.

### 2.4. Magnetic Hyperthermia of MNPs Under a Biased DC Magnetic Field

**Figure 4:**
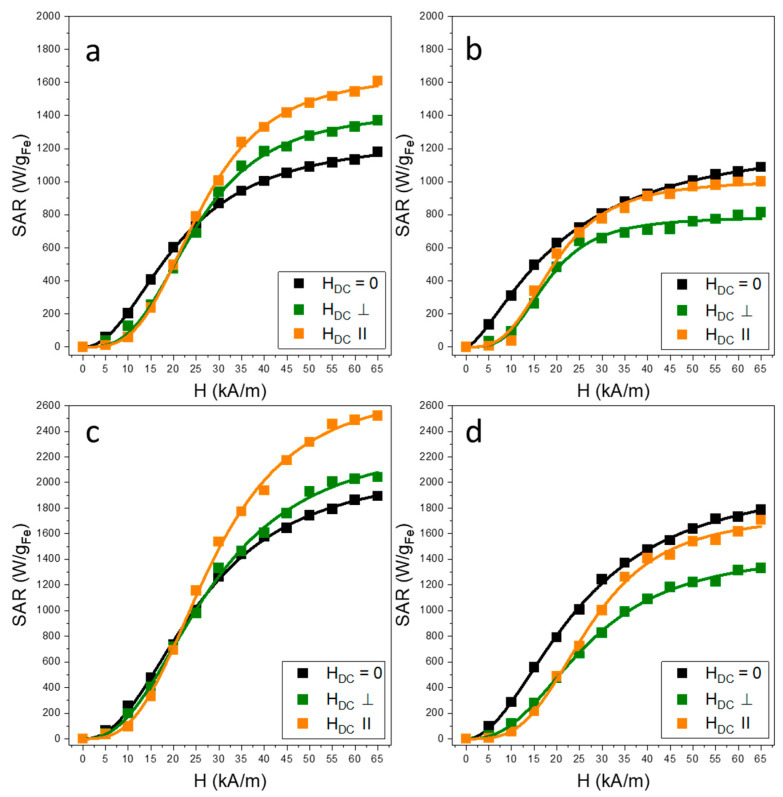
SAR dependence on the H for dicarboxylate and dendron-coated spheres (**a**,**b**) and cubes (**c**,**d**) without and with a biased DC magnetic field oriented either parallel or perpendicular to the AMF, dispersed in water at a concentration of 1 mg_Fe_/mL. The lines are the best fits using the logistic function.

The magnetic energy gained by spherical and cubic MNPs when exposed to an AMF is believed to surpass the thermal energy, leading to the creation of chains during MH experiments. However, to check and ensure the formation of these chains, a static bias H_DC_ of 10 kA/m was added to the AMF during the MH experiments (Figure S4). Aiming to confirm this, the samples containing MNPs were placed onto the TEM grid while subjected to an H_DC_ of 10 kA/m. Subsequently, they were permitted to undergo drying before their introduction into the TEM. As depicted in Figure S5, both types of MNPs are arranged predominantly in chains. The H_DC_ was applied either parallel or perpendicular to the AMF lines (Figure S4). Figure 4 illustrates the variation of SAR values with respect to H for both dendron and dicarboxylate-coated spherical and cubic MNPs.

The SAR values of MNPs are affected by the application of H_DC_. For both magnetic configurations, two distinct SAR behaviors are observed in the case of dicarboxylate-coated spherical and cubic MNPs, as shown previously by us in the case of zinc ferrite NPs [67]. Within the low H range (5–20 kA/m), SAR values are below those obtained without extra H_DC_ (Figure 4a,c). Upon entering the high H ranges (H > 30 kA/m), SAR values significantly increase compared to cases without added H_DC_ (Figure 4a,c). The parallel configuration exhibits the most pronounced enhancement of SAR. The sigmoidal relationship between SAR and H reveals a displacement of H_cHyp_ towards higher values for both magnetic configurations. Notably, both spheres and cubes exhibit a comparable H_cHyp_ (Table 2). Nevertheless, cubes display a more noticeable SAR enhancement: in the parallel configuration, SAR_max_ exhibits a rise of 620 W/g_Fe_, compared to the 370 W/g_Fe_ increase observed for spheres. As we have previously elucidated [67], the shift of H_cHyp_ is due to the interaction of the MNPs with the H_DC_. This interaction causes the MNPs to align parallel with the H_DC_ lines. Once the H exceeds 10 kA/m, the MNPs shift their orientation, leading to efficient heating. With further elevation of H under the influence of the H_DC_, which aligns the MNPs along its lines, there is an increase in the mutual attraction among the MNPs. This alignment encourages the formation of chains, facilitating the emergence of extrinsic anisotropy, which, in turn, contributes to a significant rise in SAR values. In this situation, cubic MNPs, owing to their distinctive geometry, exhibit more efficient chain association compared to spheres. As a result, the cubic MNPs release a larger amount of heat. A notable increase in SAR is observed in the perpendicular configuration within the higher range of H values, in contrast to the case where only AMF is used. SAR_max_ elevates by 220 W/g_Fe_ for cubes and 145 W/g_Fe_ for spheres. It is unclear whether chaining occurs along AFM lines in this perpendicular configuration; however, the synergistic effect of H_DC_ and H could induce the structuration of MNPs, resulting in a more coherent magnetization reversal and improved heat dissipation capabilities.

The evolution of SAR values with H for dendron-coated MNPs as a function of H_DC_ showed a distinctly dissimilar pattern (Figure 4b,d). The sigmoidal dependence of SAR over H remains consistent, yet there is a larger shift of H_cHyp_ towards a higher value as compared to dicarboxylate-coated MNPs (Table 2). For both magnetic configurations, during the low H range, the SAR values are inferior to those observed for dicarboxylate-coated MNPs under equivalent conditions (as illustrated in Figure 4). Interestingly, upon surpassing the 25 kA/m threshold for H, the SAR values almost approximate (being slightly below) those acquired without additional H_DC_ in the parallel configuration (Figure 4b,d). However, in the perpendicular configuration, there is a marked decrease in SAR values; SAR_max_ exhibits a reduction of 665 W/g_Fe_ for cubes and 485 W/g_Fe_ for spheres (Figure 4b,d).

**Figure 5:**
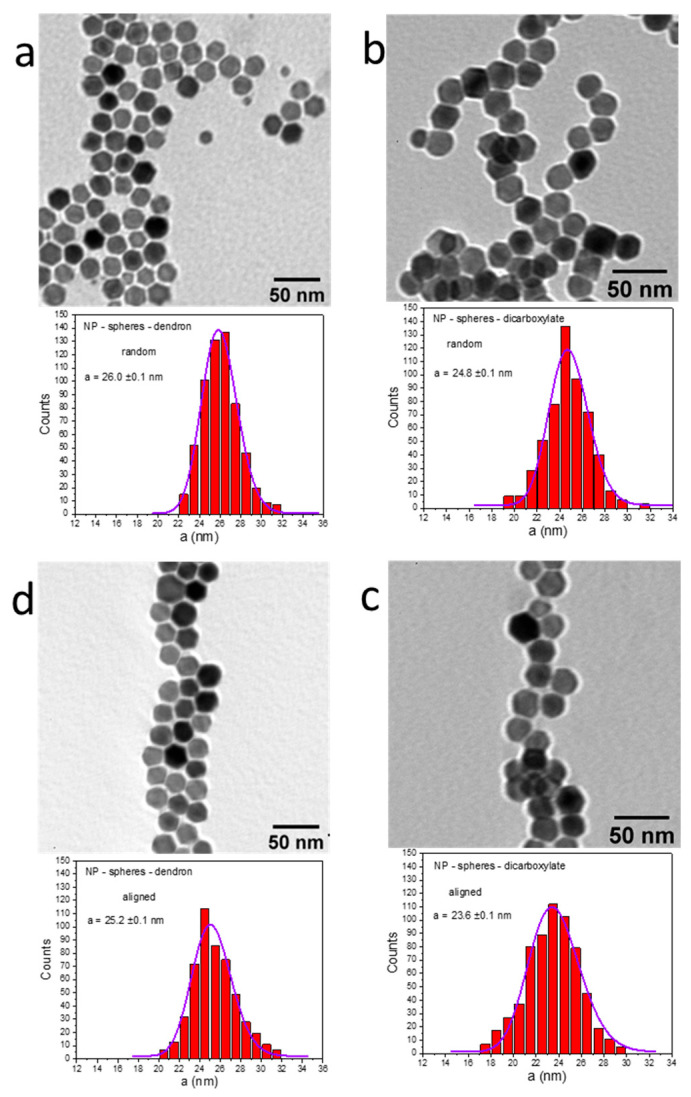
(Upper panels) TEM image of (**a**) dendron and (**b**) dicarboxylate-coated MNPs randomly distributed onto the TEM grid and of (**c**) dicarboxylate and (**d**) dendron-coated MNPs subjected to an H_DC_ of 10 kA/m during the drying process onto the TEM grid. (lower panels) The corresponding center-to-center distribution histograms are fitted to a log-normal distribution (purple lines).

Applying an H_DC_ of 10 kA/m enhances the mutual attraction among MNPs, causing them to form well-ordered structures that amplify their SAR_max_. This effect, as illustrated in Figure 4, is only significant for dicarboxylate-coated MNPs. When MNPs are surrounded by a dendron layer, the enhancement of SAR_max_ is absent. This suggests that the presence of dendrons inhibits the proximity of MNPs, likely preventing direct physical contact between them. A suitable method to confirm this phenomenon involves generating histograms of center-to-center distances from various TEM images of randomly deposited or ordered MNPs, both coated with dendrons and dicarboxylate-coated, under the influence of an H_DC_ of 10 kA/m. The research primarily focused on spherical MNPs. According to TEM images, dendron-coated spherical MNPs are individually dispersed across the carbon substrate, exhibiting an average center-to-center distance (a) of 26.0 nm (Figure 5a).

By subtracting the average diameter of 21.6 nm, the remaining difference of 4.4 nm, divided by 2, yields the dendron layer’s thickness of 2.2 nm around the magnetic core. In contrast, dicarboxylate-coated MNPs that have been transferred to water through OA oxidation tend to aggregate and form chains, displaying an average center-to-center distance of 24.8 nm (Figure 5b). After subtracting the average diameter of 21.6 nm, the difference of 3.2 nm (i.e., 1.6 nm thickness of the carboxylate layer) indicates that these dicarboxylate-coated MNPs are positioned more closely to one another, a fact also evident in the TEM images. The dipole energy can be calculated using the formula Ed~(2 × 10^7^)µ^2^/a^3^, where µ represents the magnetic moment and a is the center-to-center distance. Consequently, the dipole energy is higher for dicarboxylate-coated MNPs compared to dendron-coated ones by approximately 15%. This increase in dipole energy corresponds to an increase in SAR for dicarboxylate-coated MNPs compared to their dendron-coated counterparts, as depicted in Figure 3 and Figure 4. This difference is especially noticeable at H values exceeding 25 kA/m, which induce chain formation among the MNPs.

Subjected to an H_DC_ of 10 kA/m, the dicarboxylate-coated MNPs align themselves and draw nearer. This causes a reduction of 1.2 nm in the center-to-center spacing compared to their initial randomized arrangement (Figure 5c). The difference of 2 nm from the average diameter of 21.6 nm signifies that the organized carboxylate-coated MNPs have a 1.0 nm thick coat, as compared to 1.6 nm in the case of random arrangement. The dipole energy experiences a 17% rise compared to the random scenario. It has been demonstrated that OA is not firmly attached to the nanoparticle surface, allowing it to move freely and facilitate the nanoparticles’ approach toward each other [83]. Therefore, during MH experiments, the MNPs could come into direct physical contact and may be magnetically locked in chains. Even though the Brownian relaxation mechanism is severely limited because the magnetically aligned particles are unable to rotate once chained, the heightened anisotropy and magnetization of chains lead to an enhancement of SAR values. When examining dendronized spherical MNPs, the center-to-center separation decreases by 0.8 nm compared to the random case (Figure 5d). This decrease leads to a 10% increase in dipole energy with respect to the random case and a decrease of 22% compared to aligned dicarboxylate-coated MNPs. The difference from the average diameter of 21.6 nm is 3.6 nm, implying an interparticle separation of 1.8 nm. Given that the dendron layer has a 2.2 nm thickness, the alignment of MNPs compresses the dendron layer only by 0.4 nm, effectively acting as a spacer that prevents direct MNPs’ contact.

This distinct behavior of the MNPs in a biased DC field as a function of their coatings might be explained by the occurrence of the fanning rotation mode [84]. For MNPs organized in chains, the fanning mode provides additional heat generation in dense or interacting MNP systems, particularly when aligned chains form under an external field. It is very likely that in the case of the carboxylate-coated MNPs, the short distance between the MNPs increases their dipolar interactions and leads to a fanning rotation mode. The fanning configuration induces local anisotropy modifications, increasing the hysteresis loop area and improving heat dissipation. Moreover, in dense MNP systems, fanning mode helps maintain efficient energy dissipation even when individual nanoparticles would otherwise be in a blocked state. It can be reasoned that, even in the case of perpendicular orientation, the static bias magnetic field may lead to some degree of structuring in short-coated MNPs, potentially inducing a fanning rotation mode, which could explain the observed increase in SAR.

In the case of dendron-coated MNPs, the increased distance between individual MNPs mitigates the fanning rotation mode and thus reduces the heat released under the external AMF. For dendron-coated MNPs, heat can be generated through the Brownian relaxation mechanism. The application of an H_DC_ induces stiffness that could reduce the Brownian relaxation mechanism compared to cases where only an AMF is applied. This discrepancy could account for the slight decrease in SAR values for dendronized MNPs subjected to H_DC_ in a parallel configuration. In contrast to the parallel configuration, where MNPs complete rotations within the periods of maximum field (H + H_DC_ during the first half period and H − H_DC_ during the second half period), the perpendicular configuration limits MNPs to partial rotations within the range of +/− arctan(H/H_DC_) due to the rotating field they experience (Figure S4). As a result, Brownian heating is reduced in the perpendicular configuration, leading to a significant decrease in SAR values.

## 3. Materials and Methods

### 3.1. Materials

Iron stearate (II) (FeSt_2_) was prepared by the precipitation of sodium stearate NaSt (purity 98.8%)—purchased from TCI (Zwijndrecht, Belgium) and ferrous chloride FeCl_2_ salts—purchased from Sigma Aldrich (Diegem, Belgium) in an aqueous solution [15]. Iron stearate (FeSt_3_) was directly purchased from TCI (Zwijndrecht, Belgium, purity min 60% of stearic acid, 5.8 to 7% of iron, and maximum 10% of free acid). Organic solvents were purchased from Carlo Erba (Val-de-Reuil, France). Oleic acid (OA) was purchased from Alfa Aesar (Haverhill, MA, USA), while Sigma Aldrich (Diegem, Belgium) provided 1-octadecene, acetonitrile, ethyl acetate, and sodium periodate (NaIO_4_). Dibenzylether (DBE) and squalene were purchased from Acros Organics (Geel, Belgium). The dendron D1-2P was provided by Superbranche (Strasbourg, France).

### 3.2. Methods

#### 3.2.1. Synthesis of Spherical MNPs

A total of 2.0 mmol of commercial FeSt_3_ (1.85 g), 6.7 mmol of OA (1.90 g) (15.8 g, 19.5 mL), and 2.6 mmol of dibenzylether (0.53 g, 0.5 mL) were mixed with squalane in a two-neck round-bottom flask of 100 mL. The mixture was first heated to 120 °C (heating device temperature: 130 °C) for 60 min to dissolve the reagents in squalane. After this step, the condenser was connected to the flask, and the mixture was heated up to 330 °C (heating device temperature: 360 °C) with a 1 °C/min ramp. The mixture was refluxed at 330 °C for 60 min. The obtained black NPs suspension was cooled down to 100 °C to proceed to the washing step.

#### 3.2.2. Synthesis of Cubic MNPs

In a 100 mL two-neck round-bottom flask, 1.44 g (2.3 mmol) of FeSt_2_, 0.18 g (0.63 mmol) of OA, and 0.70 g (2.29 mmol) of NaOl with a NaOl/OA ratio of 80/20 were mixed in octadecene (11.8 g, 15 mL). The mixture was initially heated to 110 °C for 60 min to facilitate the dissolution of the reagents in the solvent. Subsequently, the condenser was coupled, and the temperature was gradually raised at a rate of 5 °C per minute until reaching 330 °C. The mixture was then refluxed at 330 °C for 60 min. Following the reflux, the resulting black nanoparticle suspension was cooled to 100 °C for the subsequent washing steps.

#### 3.2.3. Dendronization and Hydrophilization of OA-Coated MNPs

The dendronization of MNPs was carried out through a ligand exchange process between OA and the dendron, following a methodology outlined in previously published papers [14,26]. The hydrophilization of MNPs was obtained by the oxidation of OA attached to MNPs and was conducted following this protocol: a heterogeneous mixture was prepared by combining 2 mL of MNPs dispersed in hexane, 2 mL of a 1:1 volumetric mixture of acetonitrile and ethyl acetate, and 1.5 mL aqueous sodium periodate (NaIO_4_) solution (6%); the mixture underwent mechanical stirring until the MNPs completely migrated from the organic to the aqueous phase (occurred over two hours); subsequently, MNPs were separated using a permanent magnet and redispersed in water; to remove any remaining unreacted NaIO_4_ and oxidation byproducts, the MNPs were subjected to magnetic separation and redispersion in water for a minimum of three cycles.

#### 3.2.4. Characterization Techniques

Transmission electron microscopy: Transmission electron microscopy (TEM) images were acquired using a JEOL JEM-100CX II microscope (JEOL, Tokyo, Japan) operating at 80 kV, equipped with a MegaView G3 camera (Emsis, Münster, Germany) and controlled by Radius 2.1 software (Emsis). A droplet of an aqueous suspension containing 10 µg/mL of MNPs was placed onto a carbon-coated copper grid. After a 5 min incubation period, excess water was carefully removed using filter paper.

Fourier transform infrared spectroscopy: Fourier transform infrared (FT-IR) spectra were acquired using a TENSOR II instrument (Bruker Optics Inc., Billerica, MA, USA) configured in attenuated total reflectance mode. The platinum attenuated total reflectance (ATR) accessory, featuring a single reflection diamond ATR, was employed for the measurements. The spectral recordings were performed within the range of 400 to 4000 cm^−1^, employing a resolution of 2 cm^−1^ and accumulating 64 scans per powder sample, obtained by removing water through a rotary evaporator.

Magnetic characterizations: The magnetization curves of the MNPs (powder) were measured using a Cryogenic Limited (London, UK) vibrating sample magnetometer (VSM), operating at both 4 K and 300 K from 0 to ±4 T. Powder samples were used.

Magnetic hyperthermia: For the MH experiments, we utilized the EasyHeat 0224 power supply station from Ambrell (Scottsville, NY, USA). This equipment operates within a frequency range of 100–400 kHz, enabling the generation of magnetic fields with intensities as high as 65 kA/m [85]. The heating capabilities of MNPs were assessed using the specific absorption rate (SAR), which was calculated following the methodology outlined in the Supplementary Information Section S2.

## 4. Conclusions

We investigated the role of the coating shell on the magnetic heating properties of spherical and cubic MNPs coated with dendron and short linear dicarboxylate molecules with/without an applied external bias DC magnetic field. Both spherical and cubic MNPs synthesized by the thermal decomposition technique exhibit a magnetite composition and a well-defined shape. Owing to their higher saturation magnetization, the cubes, either dendron or dicarboxylate-coated, display higher SAR values as compared to spheres over the entire H range. The dendron shell around MNPs was shown to reduce the strength of dipolar interactions among them, resulting in two distinct magnetic heating behaviors over the H range. In particular, at low H range (H × f < 9 × 10^9^ A·m^−1^·s^−1^), the dendronized MNPs heat up more effectively than dicarboxylate-coated MNPs. On the contrary, at high H range (H × f > 9 × 10^9^ A·m^−1^·s^−1^), the situation is reversed with the dicarboxylate-coated MNPs heating better than dendronized ones.

The application of an external bias DC magnetic field, either perpendicular or parallel to AMF lines, reduces the SAR values at low H range for both types of dendron and dicarboxylate-coated MNPs with respect to the case where only AFM is used. In the high H range, the effects of the DC magnetic field in both orientations are opposite for the two types of coatings. SAR of dicarboxylate-coated MNPs is enhanced, with the parallel configuration producing the highest effect. On the contrary, the SAR of dendronized MNPs is decreased in the presence of an external bias DC magnetic field. This time, the perpendicular configuration produces the largest drop in SAR values.

This work illustrates the fact that not only the intrinsic properties of MNPs are important, but also their coating drives their assembling towards favorable or unfavorable interactions for enhancing MH performances.

## Figures and Tables

**Table 1:** Magnetic hysteresis parameters of spherical and cubic MNPs.

Sample	5 K				300 K			
	M_s_ (emu/g)	H_c_ (kA/m)	M_r_ (emu/g)	M_r_/M_s_	M_s_ (emu/g)	H_c_ (kA/m)	M_r_ (emu/g)	M_r_/M_s_
Spheres	72.6	40	25.7	0.35	64.6	9	18.0	0.28
Cubes	90.9	35	32.7	0.36	72.9	9	23.6	0.32

**Table 2:** Fitting results of SAR evolution with AC magnetic field amplitude.

MNPs	H_DC_	H_cHyp_ (kA/m)	SAR_max_ (W/g_Fe_)	n
Spheres-dicarboxylated	0	13.23	1285	2.13
	II	21.38	1655	3.28
	ꓕ	19.82	1430	3.05
Spheres-dendron	0	6.94	1270	2.27
	II	15.73	1010	3.11
	ꓕ	14.78	785	3.35
Cubes-dicarboxylated	0	17.05	2130	2.27
	II	22.46	2750	2.43
	ꓕ	19.59	2350	2.97
Cubes-dendron	0	14.22	2045	1.98
	II	22.51	1750	3.32
	ꓕ	19.25	1465	2.55

## Data Availability

The original contributions presented in the study are included in the article/Supplementary Material; further inquiries can be directed to the corresponding author.

## Supplementary Materials

The following supporting information can be downloaded at https://www.mdpi.com/article/10.3390/ijms26199324/s1.
