# Supplementary material for: Comparative Influence of Dendron and Dicarboxylate Coatings on the Hyperthermia Performances of Cubic and Spherical Magnetic Nanoparticles
Source: Int J Mol Sci. 2025 Sep 24;26(19):9324. doi: 10.3390/ijms26199324 (PMC12525091; doi:10.3390/ijms26199324)
Supplement: Supplementary file 1 [file ijms-26-09324-s001.zip › ijms-3874065-supplementary.pdf]

Supplementary Materials

Comparative Influence of Dendron and Dicarboxylate Coatings on the Hyperthermia Performances of Cubic and Spherical Magnetic Nanoparticles

Cristian Iacovita [1], Constantin Mihai Lucaciu [1,*], Barbara Freis [2], Céline Kiefer [2] and Sylvie Bégin-Colin [2,3]

[1] Department of Pharmaceutical Physics-Biophysics, Faculty of Pharmacy, Iuliu Hatieganu University of Medicine and Pharmacy, 6 Pasteur St., 400349 Cluj-Napoca, Romania; cristian.iacovita@umfcluj.ro

[2] UMR CNRS-UdS 7504, Institut de Physique et Chimie des Matériaux, CNRS, Université de Strasbourg,
23 Rue du Loess, BP 43, 67034 Strasbourg, France; bcdfreis@gmail.com (B.F.); celine.kiefer@ipcms.unistra.fr (C.K.); sylvie.begin@ipcms.unistra.fr (S.B.-C.)

[3] UMR CNRS-UdS 7515, Institut de Chimie et Procédés pour l'Energie, l'Environnement et la Santé, CNRS, Université de Strasbourg, 25 Rue du Becquerel, BP 43, 67087 Strasbourg, France

* Correspondence: clucaciu@umfcluj.ro; Tel.: +40-744-647-854

S1. The structure of dendrons:

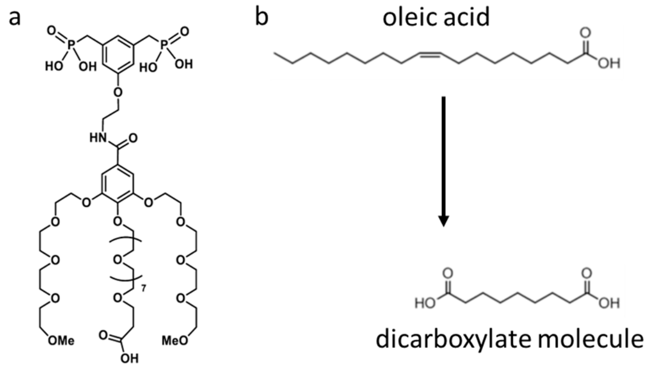

Figure S1. Chemical structure of dendron molecules developed at IPCMS and provided by the start-up Superbranche. b. The oxidation of oleic acid in a small dicarboxylate molecule.

S2. Magnetic hyperthermia:

The MH system is equipped with an 8-turn coil with an internal diameter of 2.5 mm and a total length of 40 mm. The coil inductance was calculated from its geometry and the resonance frequency of the circuit in which it was introduced and was determined as 10^{-6} H. The effective frequency and voltage values on the coil were monitored with a digital oscilloscope PeakTech 1170 (PeakTech Prüf- und Messtechnik GmbH, Ahrensburg, Germany), operating up to 250 MHz, and for the specified coil was 355 kHz. The H calibration was performed by using a copper wire with a 10 mm diameter, surrounding the vial in which the samples were introduced as a magnetic probe, and measuring the induced electromotive force by using the oscilloscope, as described in detail in our previous work [1]. The samples consisted of a 0.50 mL volume of MNPs suspended in water at an iron concentration of 1 mg_{Fe}/mL determined by inductively coupled plasma–atomic emission spectrometry. The temperature was assessed using a fiber optic sensor, placed in the middle of the sample, to provide the temperature values at one-second intervals. The MH measurements were performed in a non-adiabatic environment. To ensure a constant and physiologically relevant temperature, the test samples were placed in thin tubing with circulating water at 37 °C, thus thermally isolating them from the coil and setting 37 °C as the initial temperature for each experiment.

The temperature changes ΔT versus time curves (Figures S1 and S2) have been fitted with the Box–Lucas equation:

$$\Delta T = \frac{S_m}{k}\left(1 - e^{-k(t-t_0)}\right) \qquad \text{(S1)}$$

where the fitting parameters S_m and k are the initial slope of the heating curves and the constant describing the cooling rate, respectively. Thus, SAR can be calculated as:

$$SAR = \frac{c\, m\, S_m}{m_{Fe}} \qquad \text{(S2)}$$

where c is the colloid-specific heat (the MNPs contribution to the specific heat being negligible in our case) and was approximated with the c of water, and m = ϱV is the mass of colloid, taken as the product between the density and the volume (ϱ = 0.997 g/cm^3, c = 4186 J/kgK). Before each measurement, the liquid samples were sonicated for 10 seconds to ensure good colloidal dispersion over the entire aqueous volume. Each SAR value is the mean of three measurements performed on three different samples.

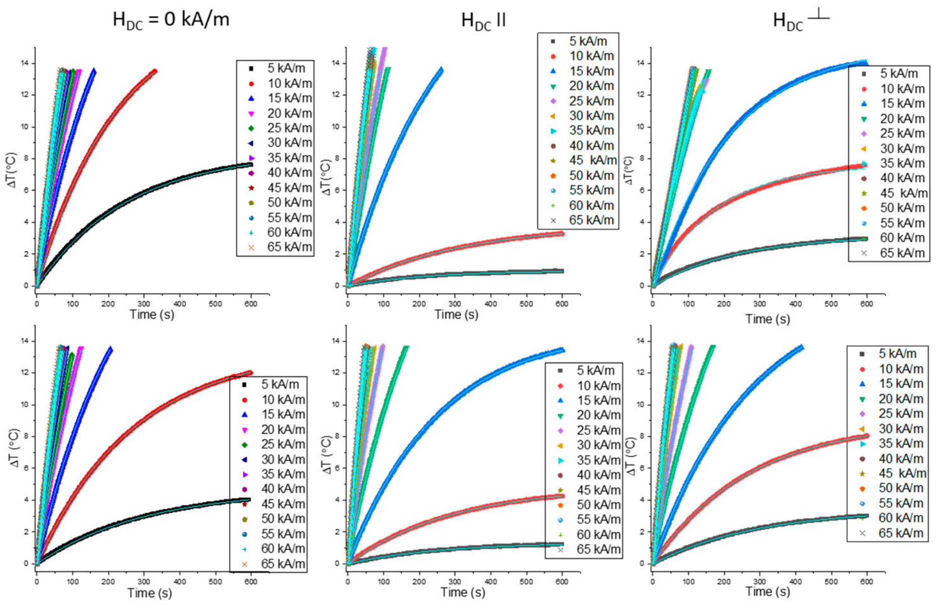

Figure S2. The temperature change ΔT versus time curves fitted with the Box–Lucas equation (blue curves) of spherical MNPs coated with dendrons (upper panels) and uncoated (lower panels), dispersed in water at an iron concentration of 1.00 mg_{Fe}/mL, recorded as a function of H (5– 65 kA/m, step of 5 kA/m) at a frequency of 355 kHz in the three configurations concerning the applied H_{DC}.

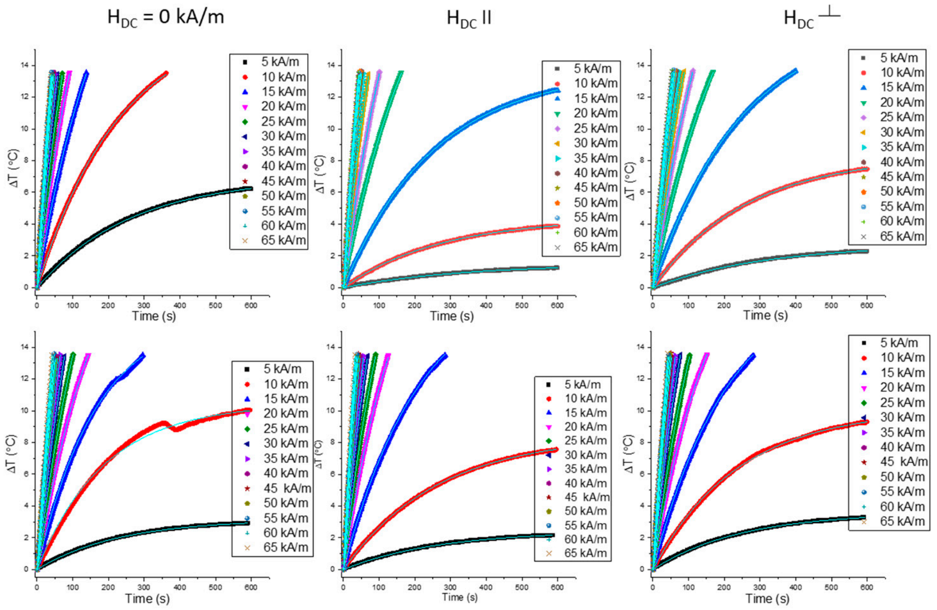

Figure S3. The temperature change ΔT versus time curves fitted with the Box–Lucas equation (blue curves) of cubic MNPs coated with dendrons (upper panels) and uncoated (lower panels), dispersed in water at an iron concentration of 1.00 mg_{Fe}/mL, recorded as a function of H (5– 65 kA/m, step of 5 kA/m) at frequency of 355 kHz in the three configuration concerning the applied H_{DC}.

Magnetic hyperthermia under a static DC magnetic field: The samples containing suspensions of MNPs in water were placed in the middle of the 8-turn coil of the EasyHeat 0224 power supply station. At the same time, an H_{DC} was created by using two neodymium magnets of 3 cm side length, placed above and below the coil for the parallel configuration and on one side and the opposite side of the coil for the perpendicular configuration (Figure S4 – left). The distance between the two neodymium magnets was varied to obtain H_{DC} of the desired intensity (10 kA/m) at the sample level within the 8-turn coil.

The magnetic induction was measured with a Brockhaus Gaussmeter BGM 101 (Dr. Brockhaus Messtechnik GmbH & Co. KG, Germany). According to the calibration curve below (Figure S4 – middle), the magnetic field was nearly constant within the central ~ 1 cm^3 region between the magnets.

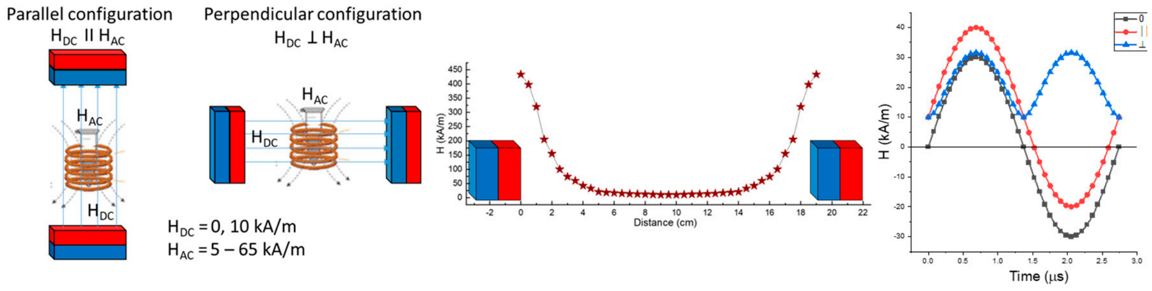

Figure S4. Experimental setup to superpose an H_{DC} over the AMF during MH measurements (left pannels). Calibration curve of the static magnetic field created by two neodymium magnets separated by 19 cm, showing a field intensity of approximately 10 kA/m in the middle region (middle panel). The time evolution of the magnetic field strength H within the coil over one period, for three different configurations: without a superimposed permanent magnetic field, and with a permanent magnetic field applied either parallel or perpendicular to the alternating field (right panel).

S3. Fitting the SAR dependence on H:

The sigmoidal evolution of our experimental SAR data with H was well fitted (R^2 > 0.999) phenomenologically with a simple logistic function:

$$SAR = SAR_{max} \frac{\left(\frac{H}{H_{cHyp}}\right)^n * \propto}{1 + \left(\frac{H}{H_{cHyp}}\right)^n * \propto} \qquad (S3)$$

with:

$$\propto = \frac{n+1}{n-1} \qquad (S4)$$

where the SAR_{max} is the saturation value of the SAR, H_{cHyp} - the hyperthermia coercive field, the value of the H for which the function presents the highest slope or the H at which the first derivative of SAR against H presents a maximum, and the exponent n – which indicates how steep the dependence of SAR on H is. The values of these parameters for all four types of MNPs are provided in Table 2 in the main text.

S4. TEM analysis:

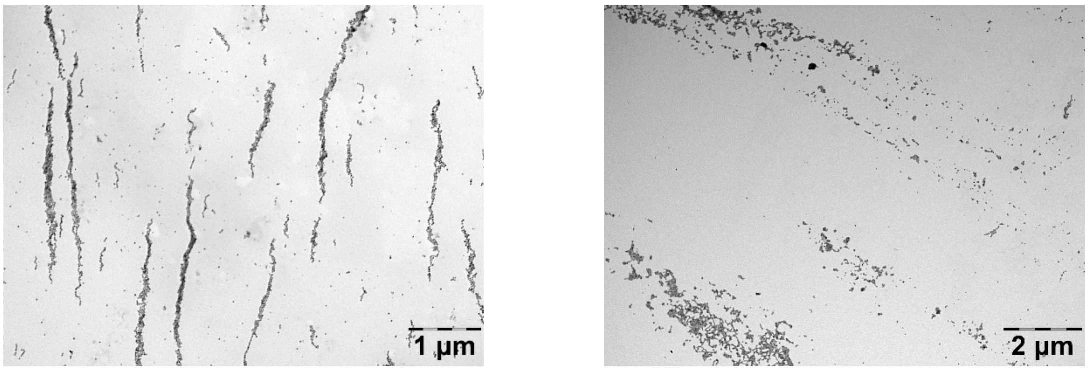

Figure S5. A typical example of a TEM image of spherical (left) and cubic (right) MNPs placed onto the TEM grid while subjected to an H_{DC} of 10 kA/m during the drying process.

References:

1. Iacovita, C.; Stiufiuc, R.; Radu, T.; Florea, A.; Stiufiuc, G.; Dutu, A.; Mican, S.; Tetean, R.; Lucaciu, C.M. Polyethylene glycol-mediated synthesis of cubic iron oxide nanoparticles with high heating power. *Nanoscale Research Letters* **2015**, *10*, 1–16. https://doi.org/10.1186/s11671-015-1091-0
